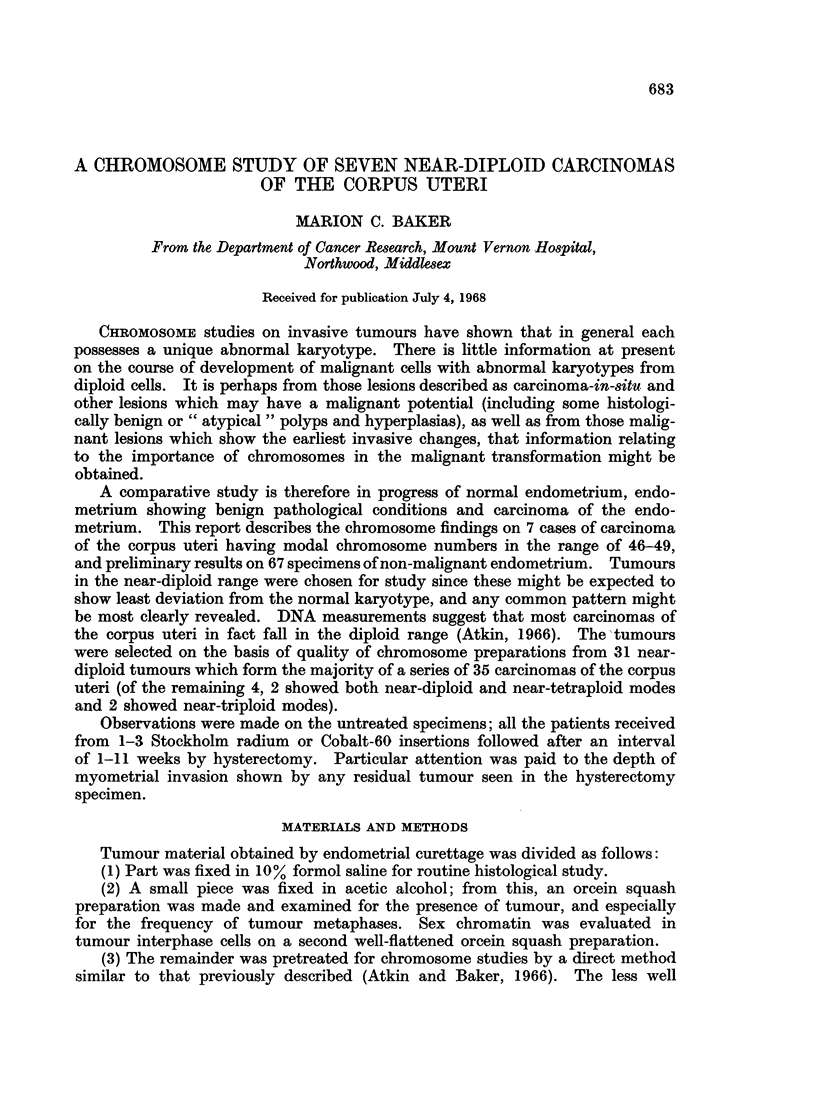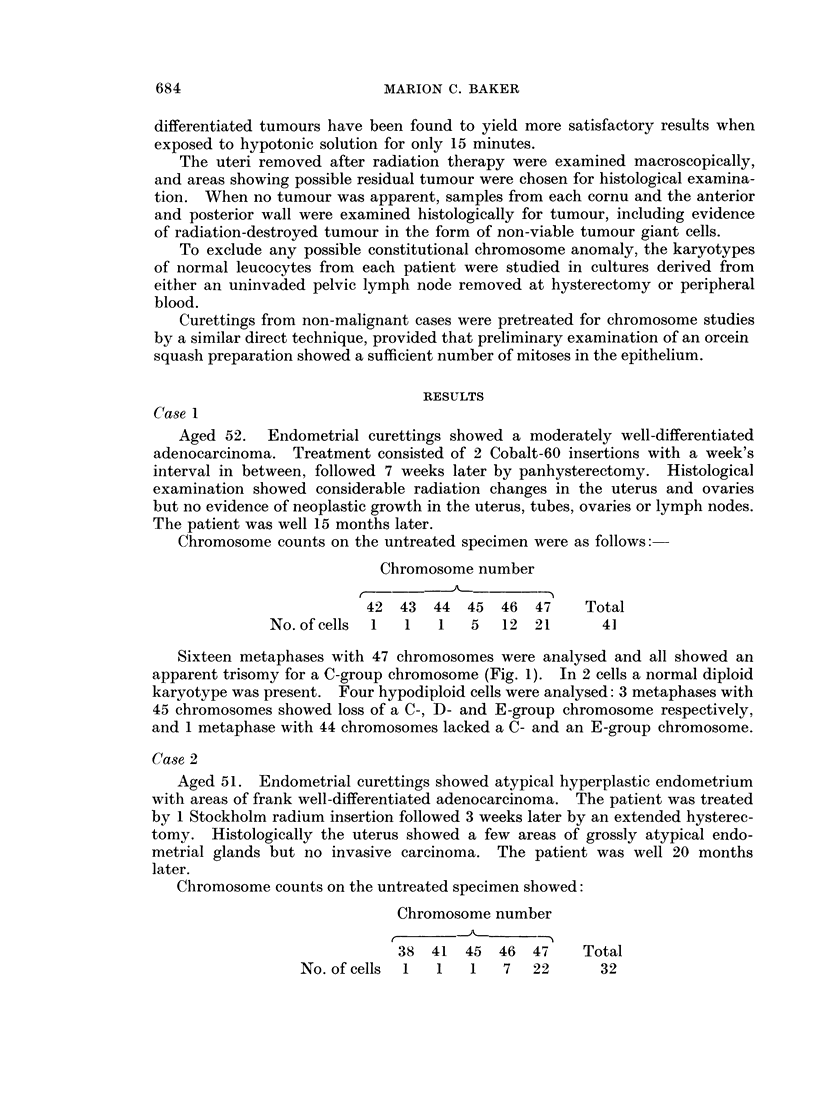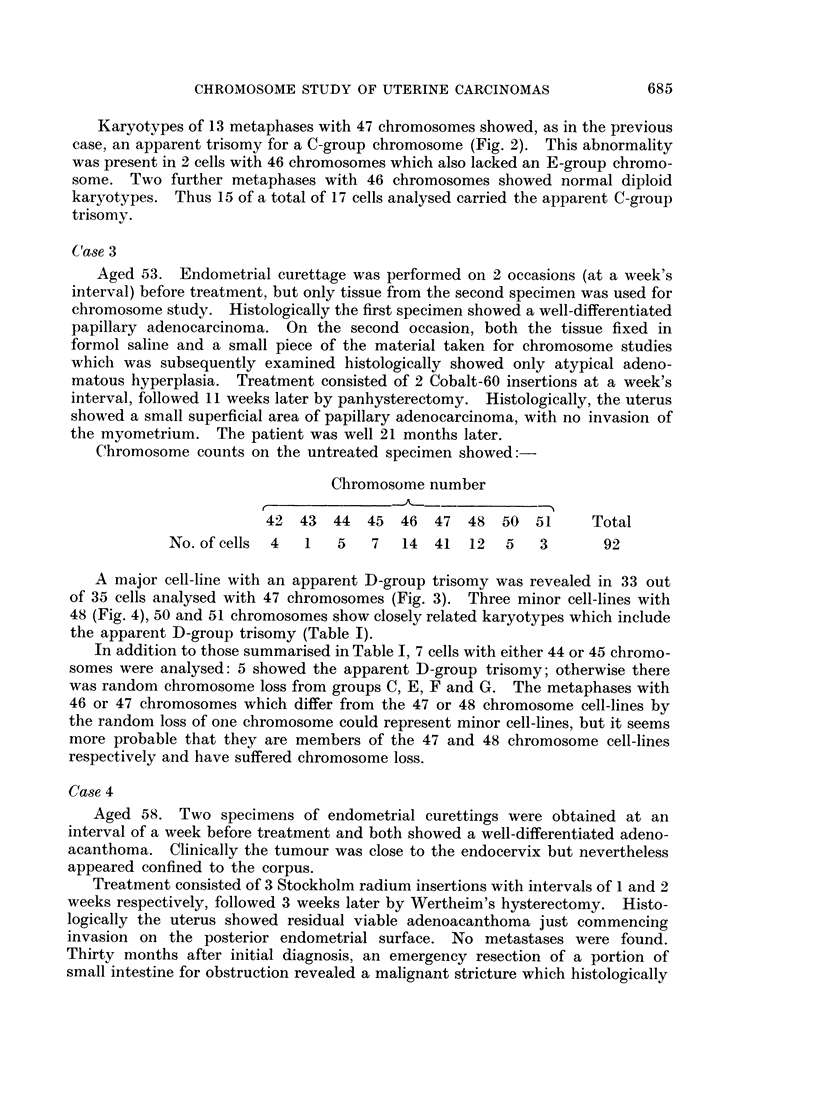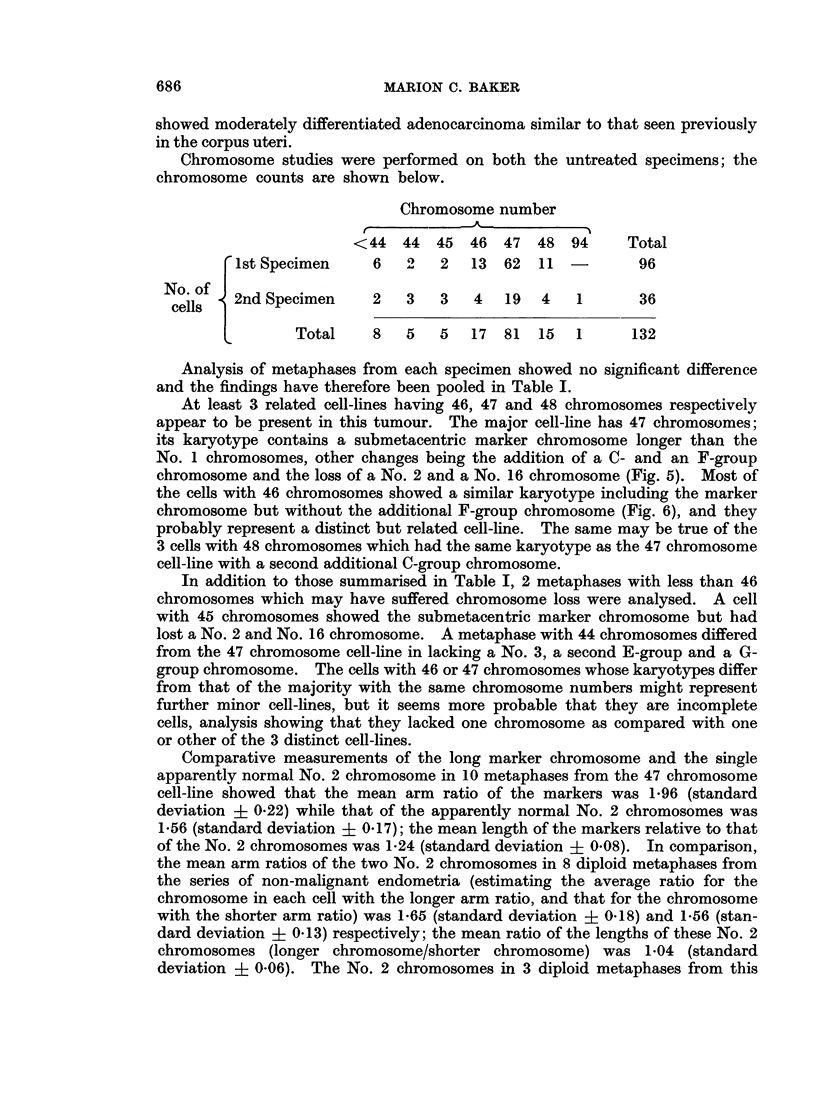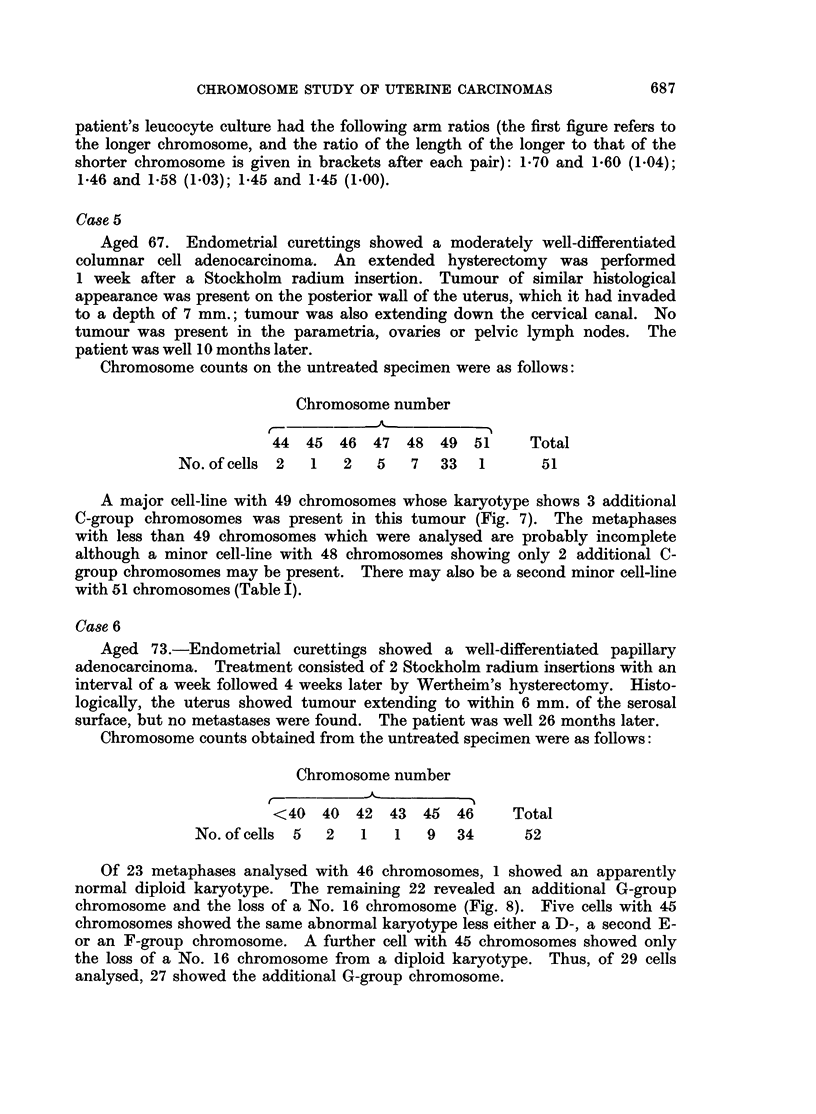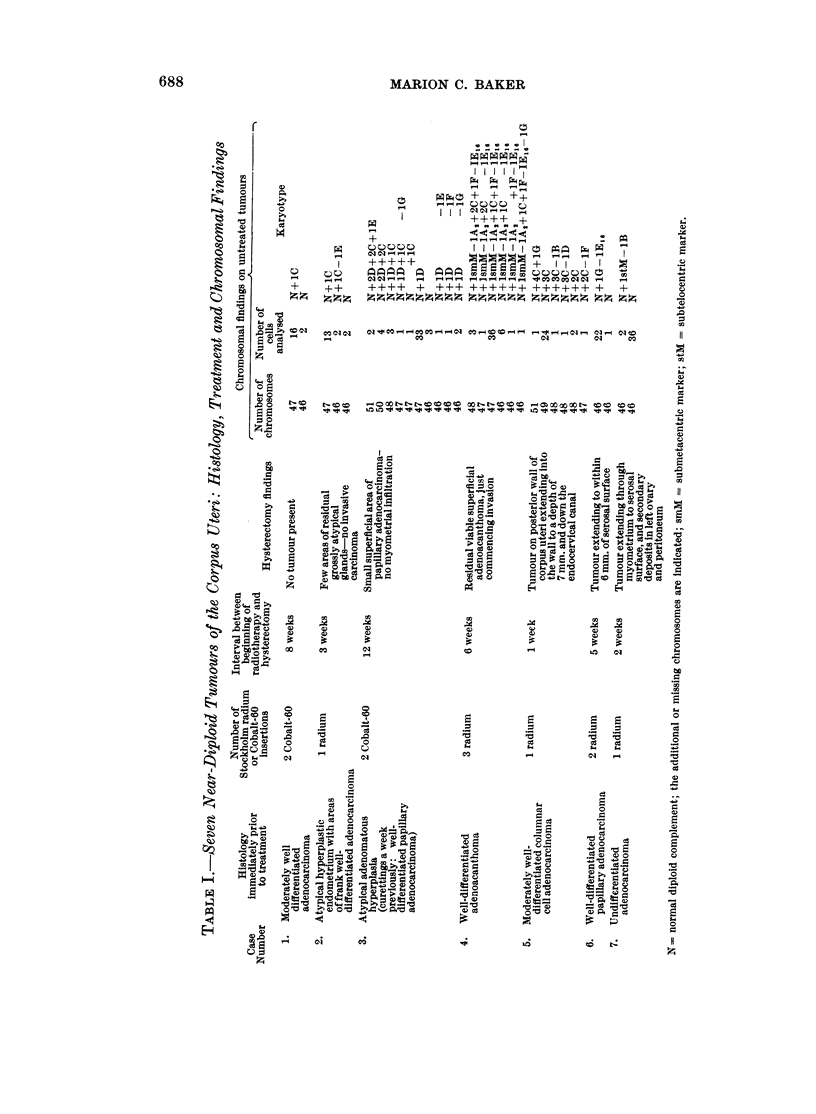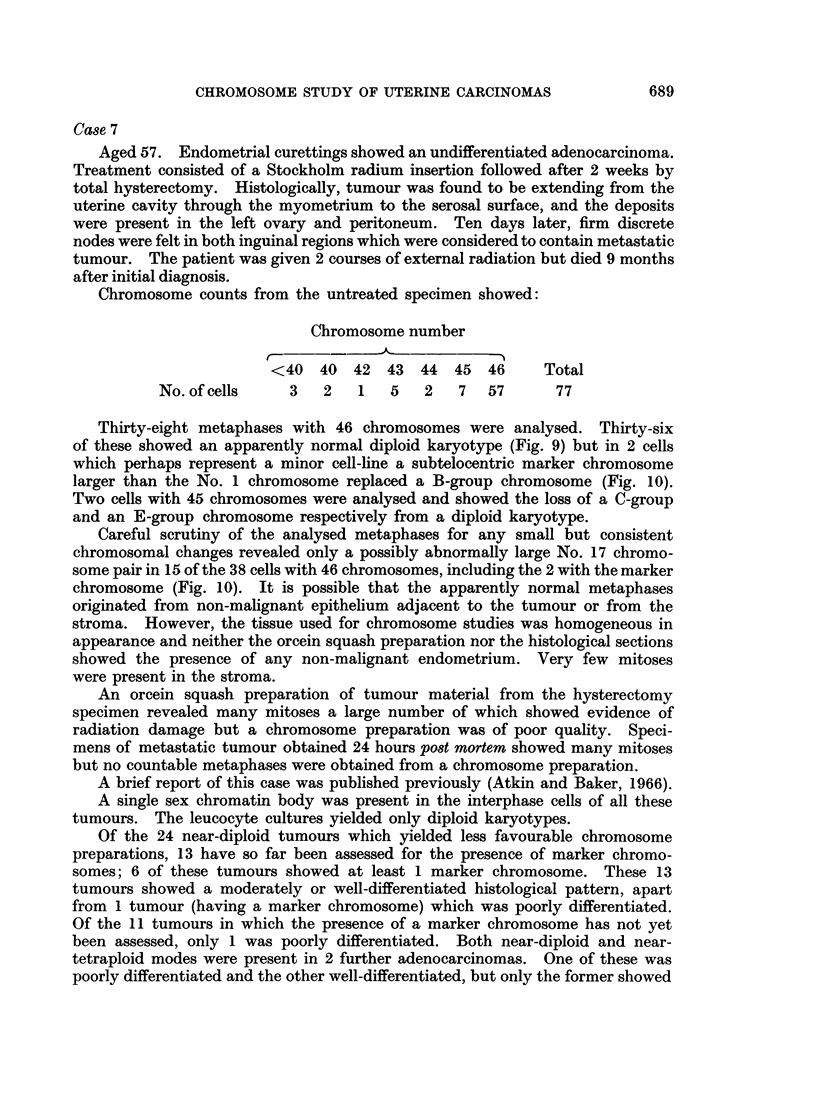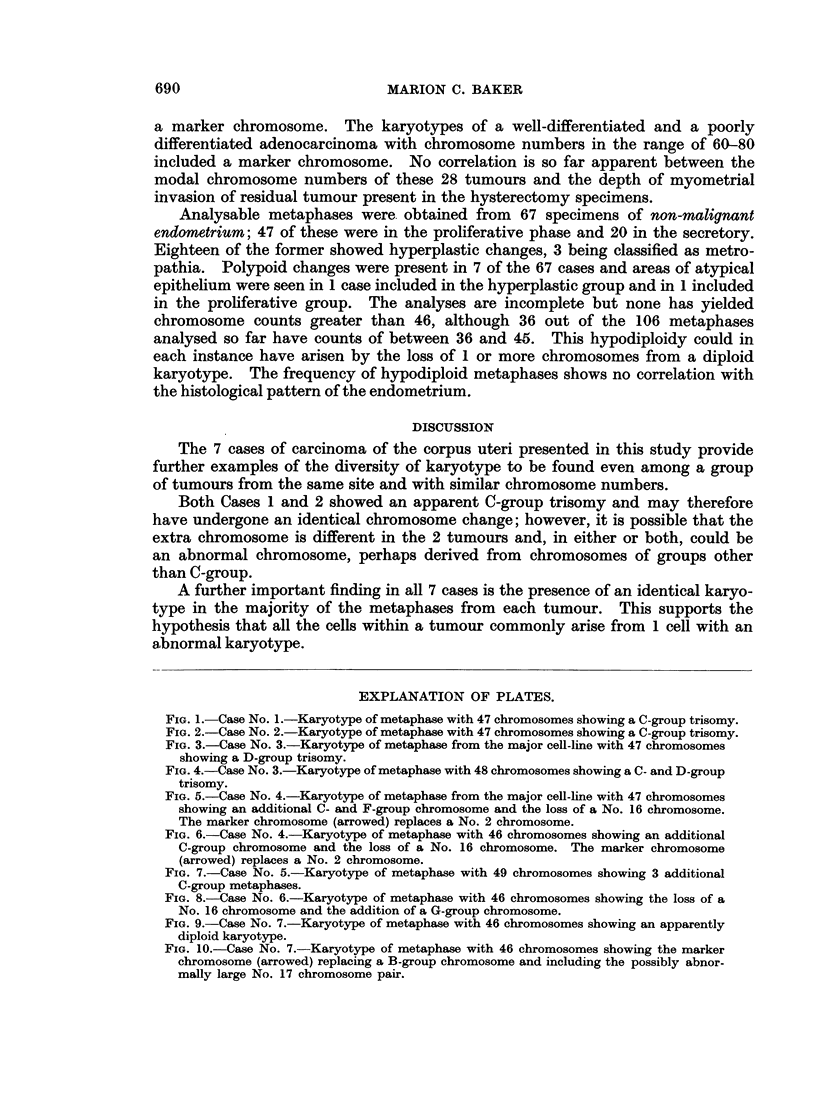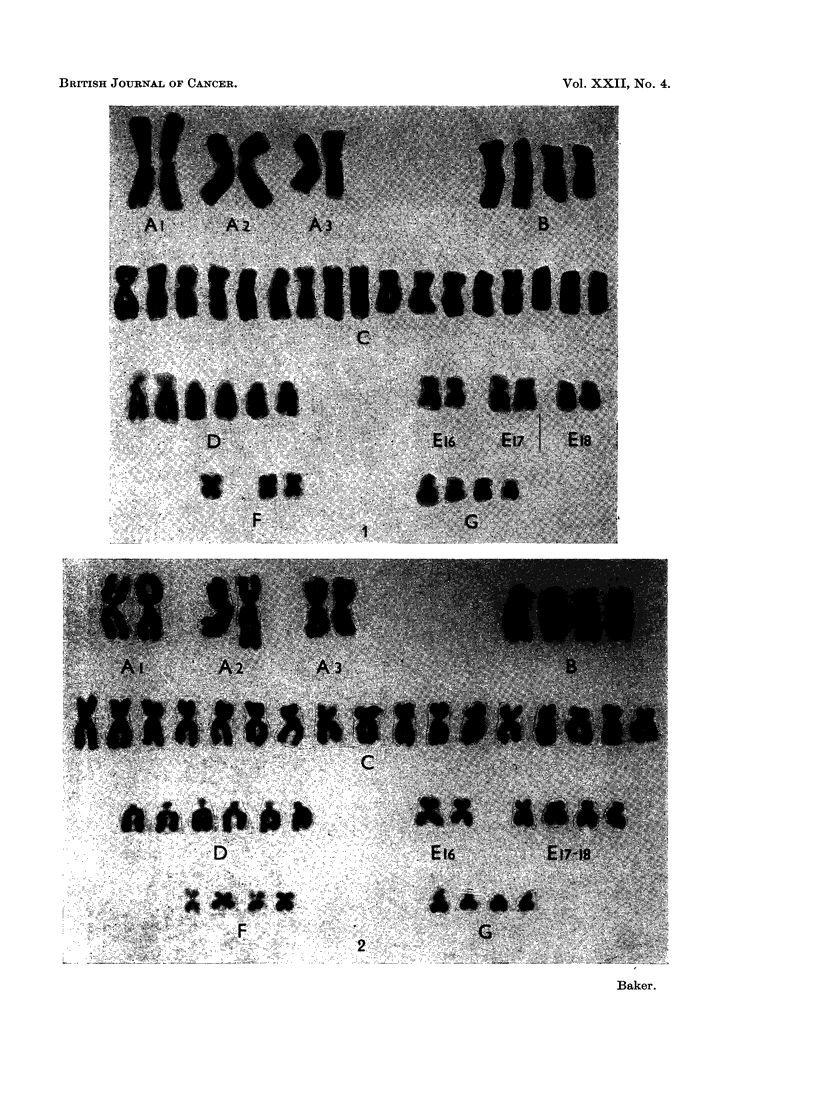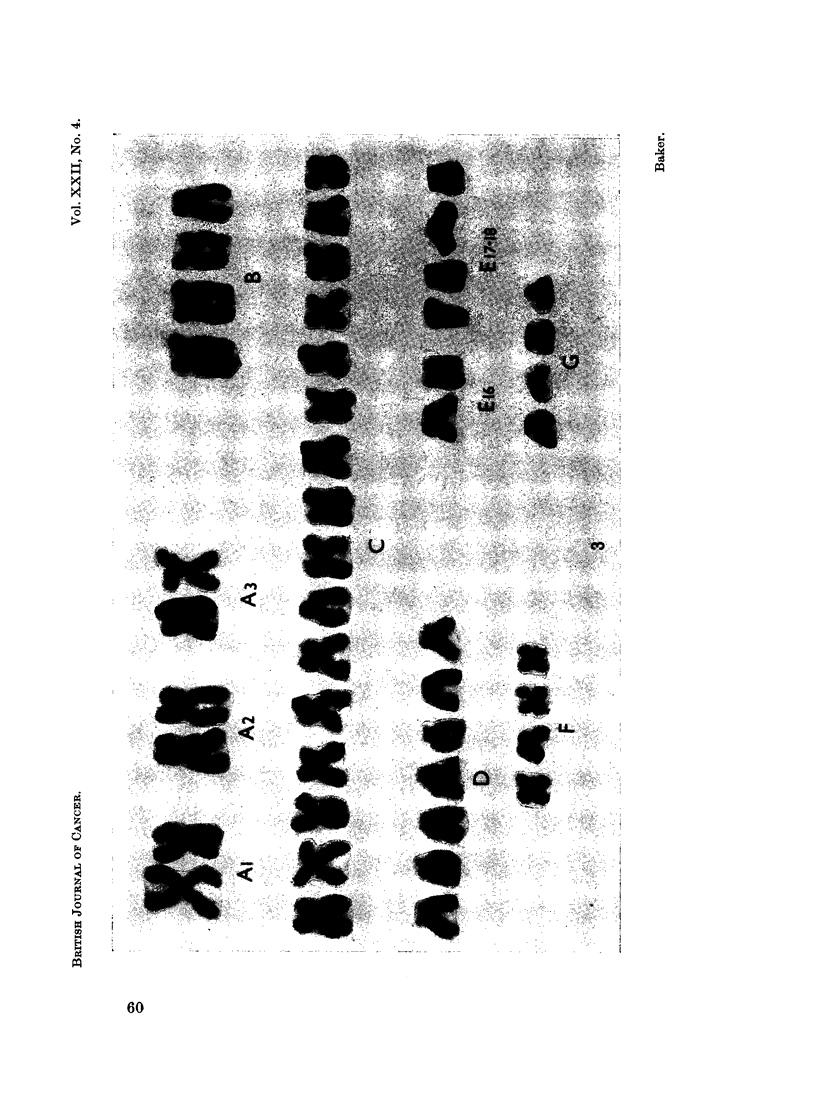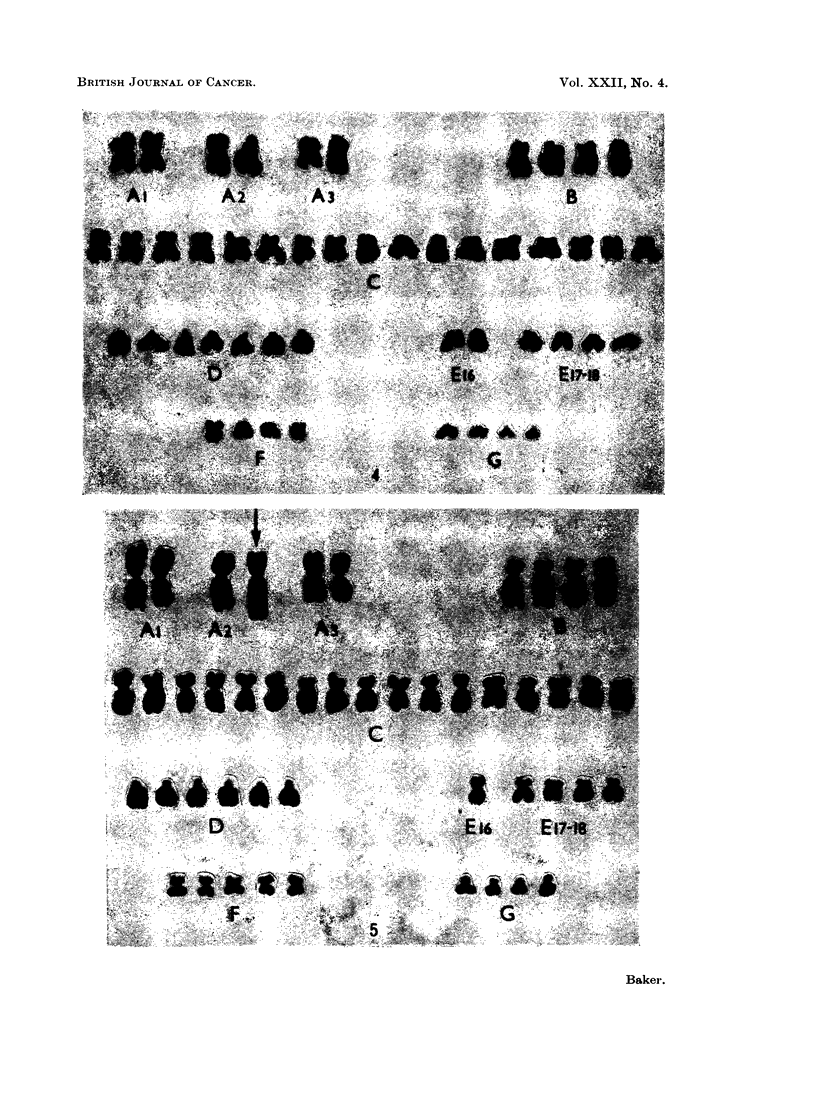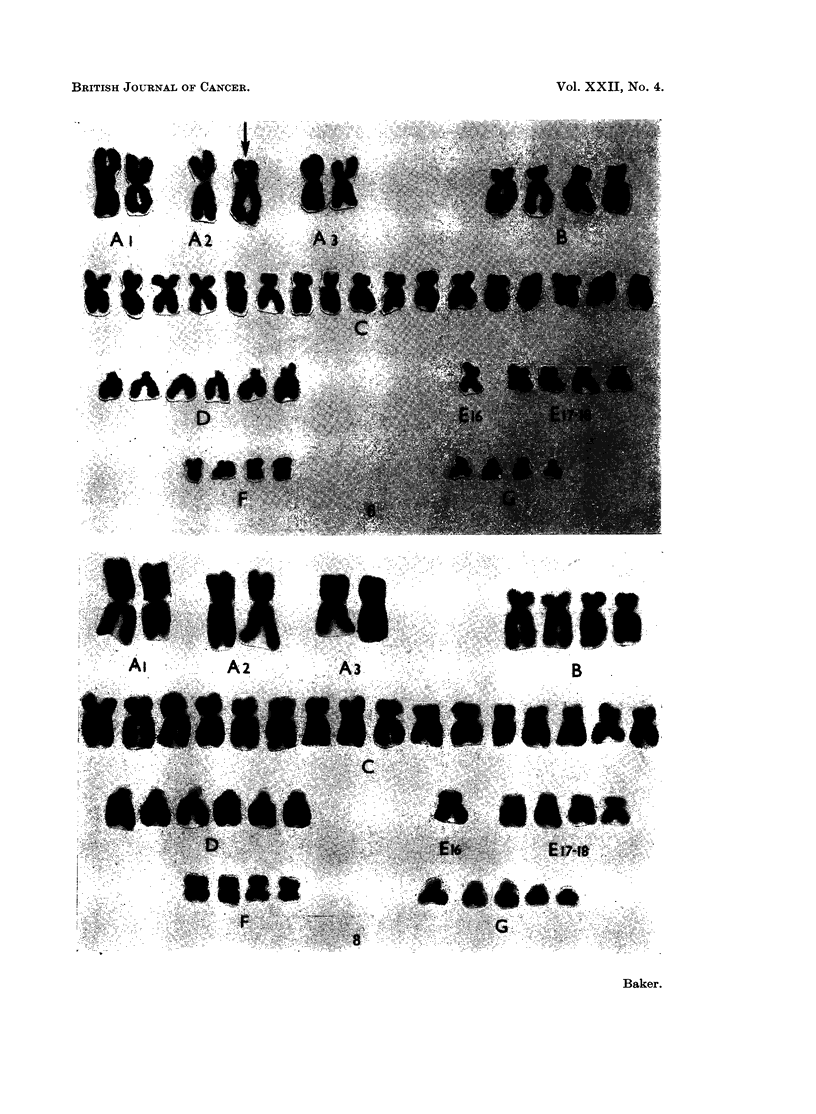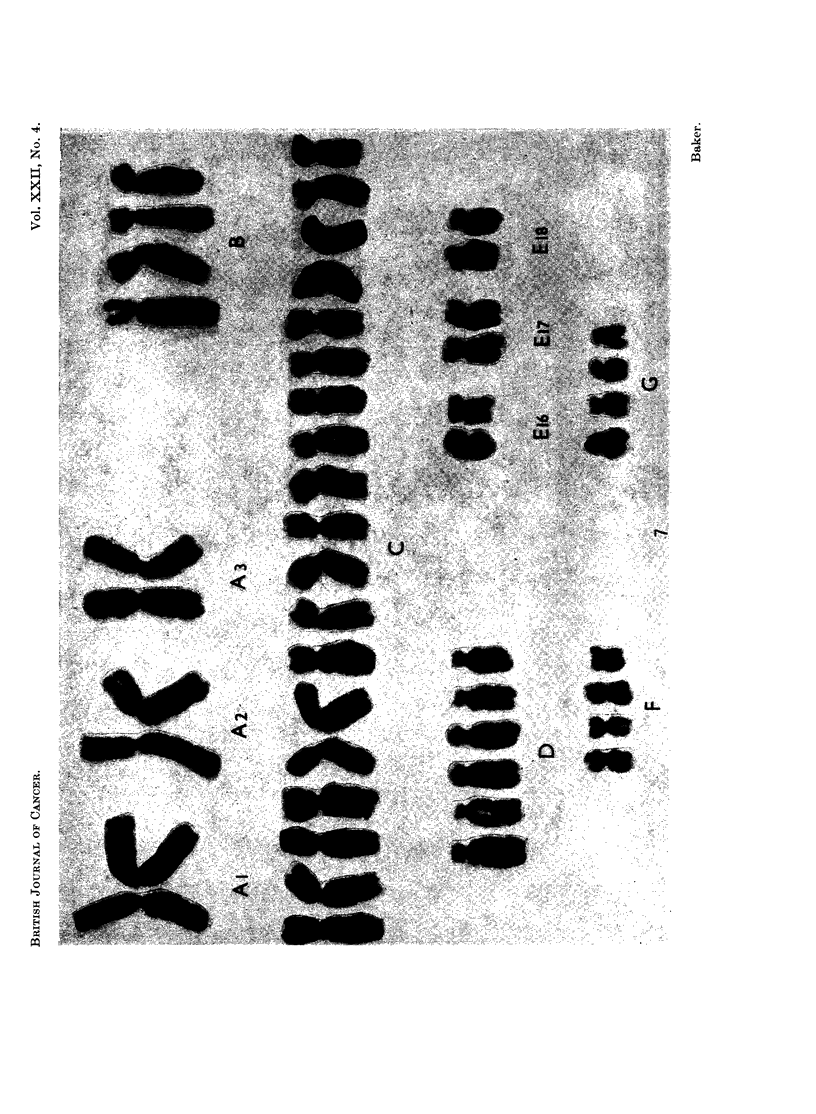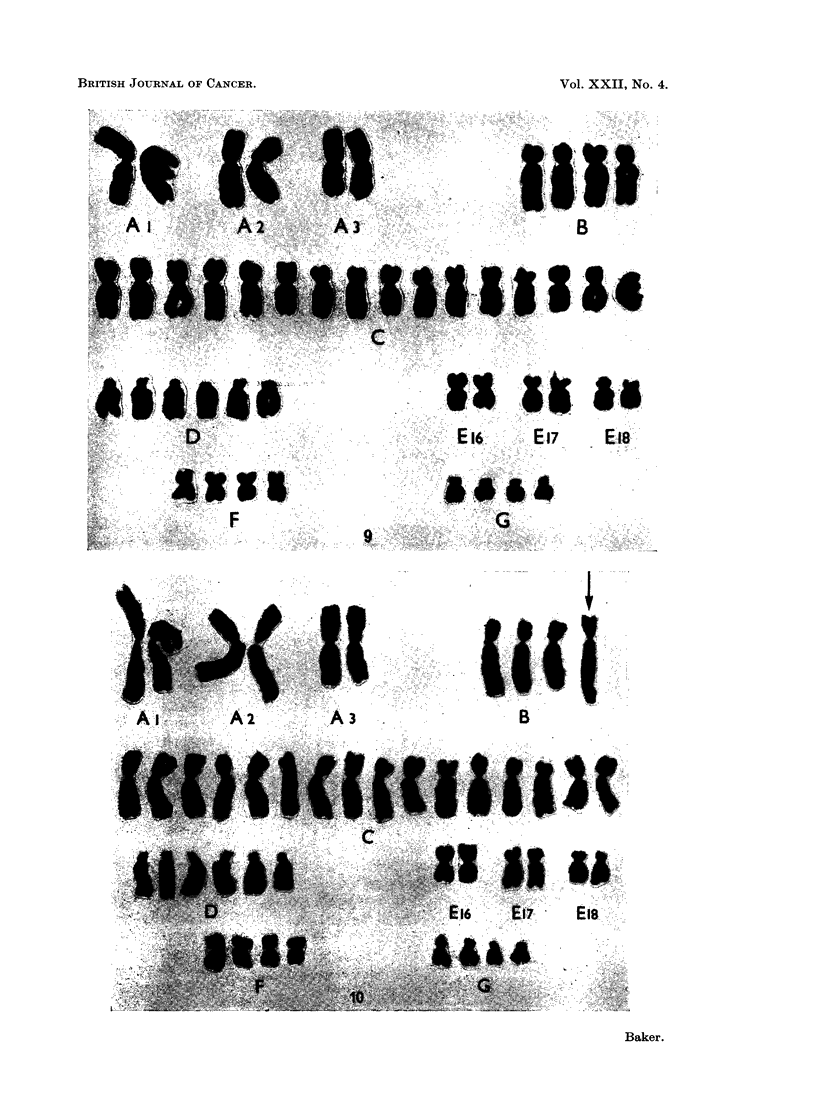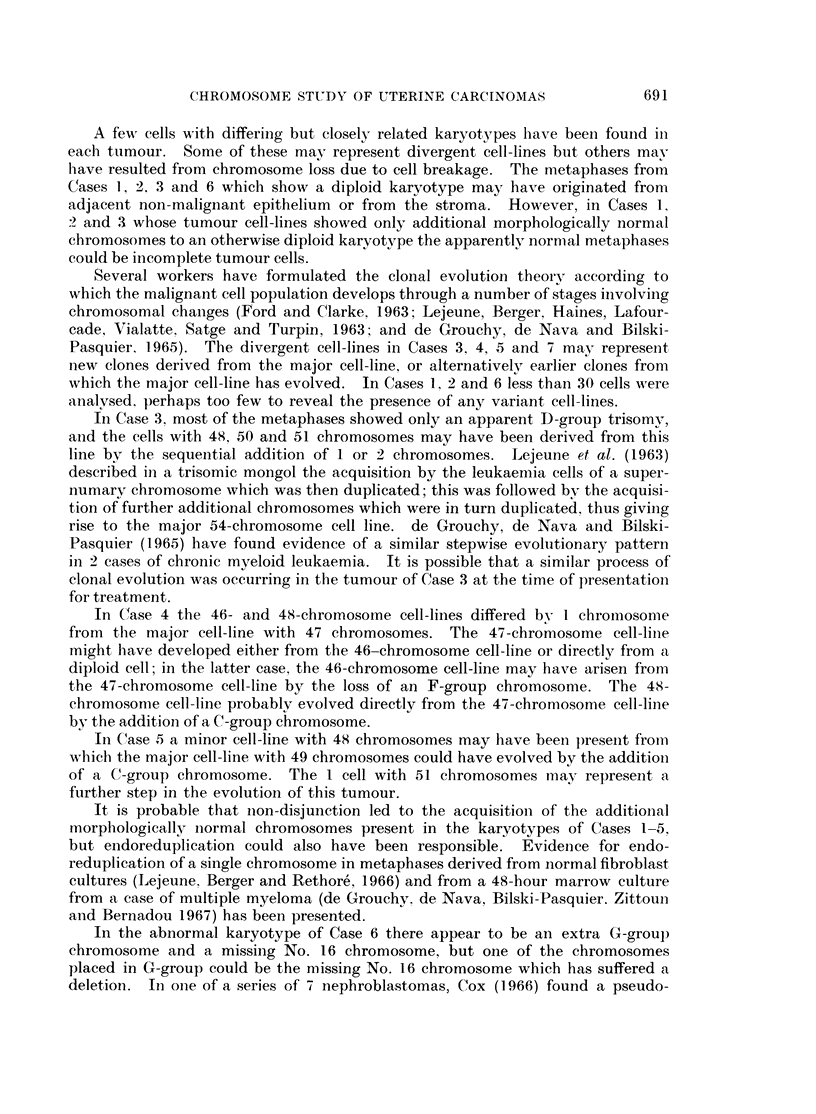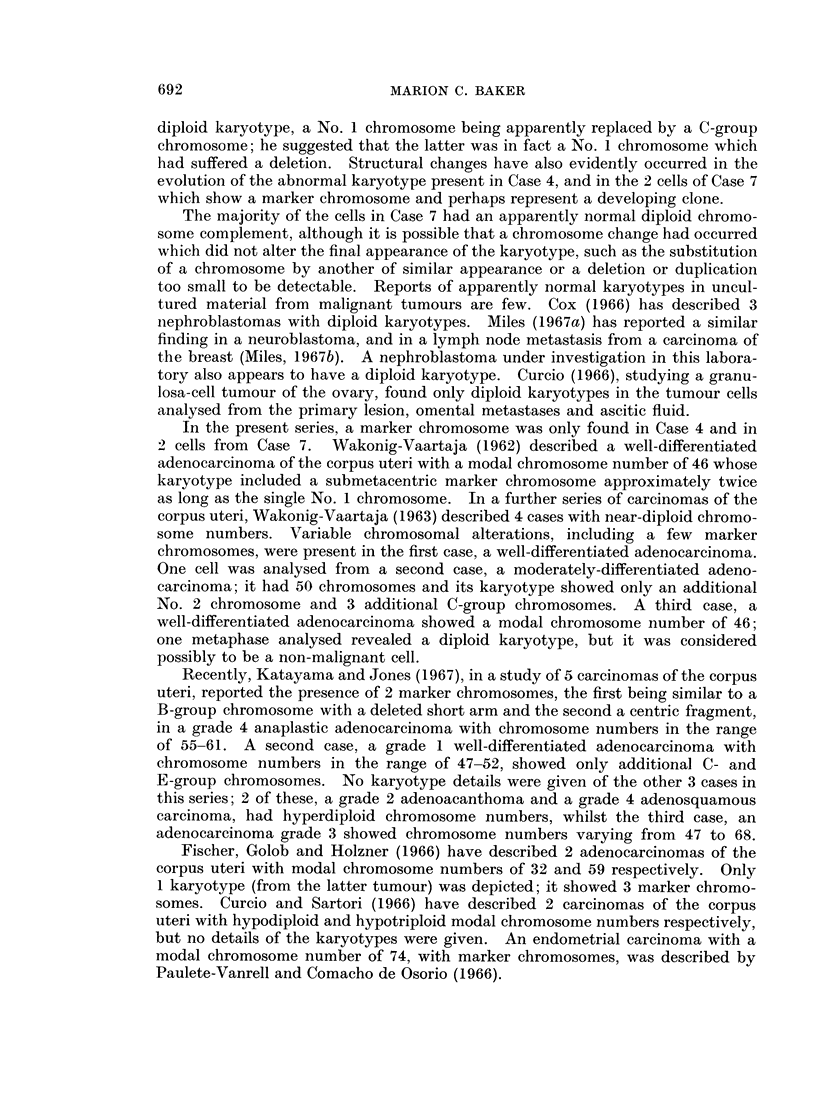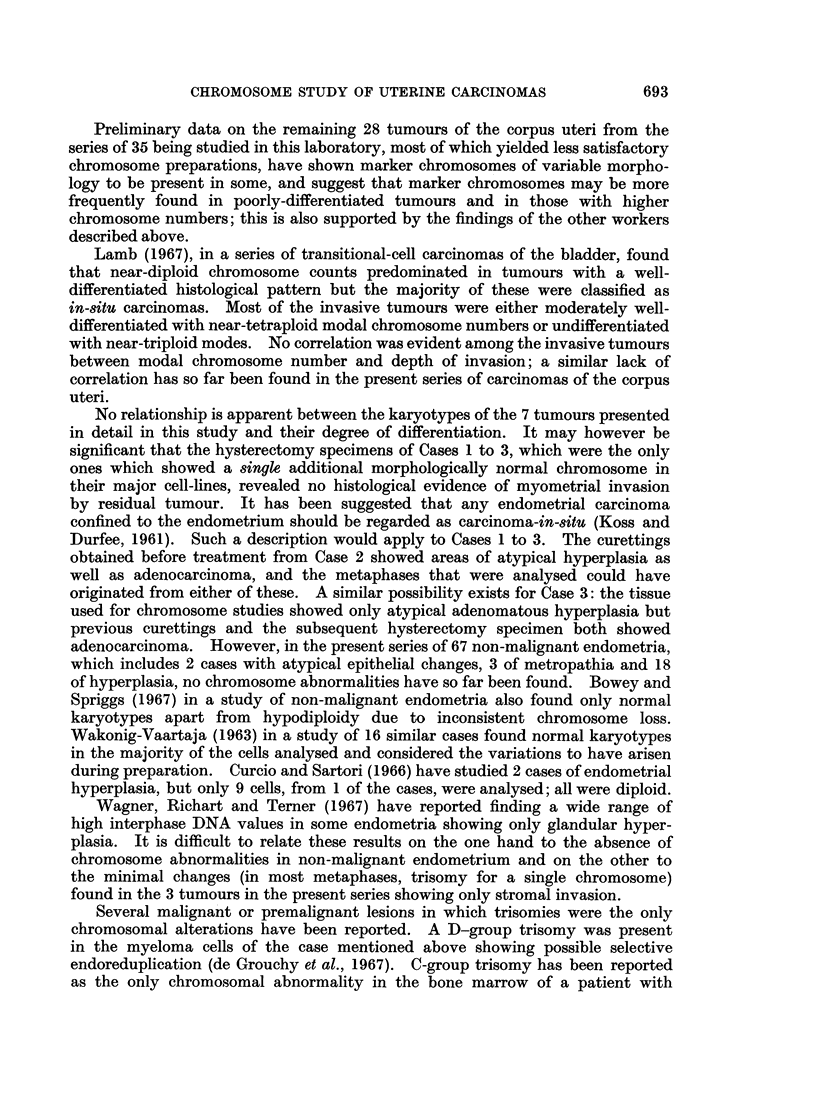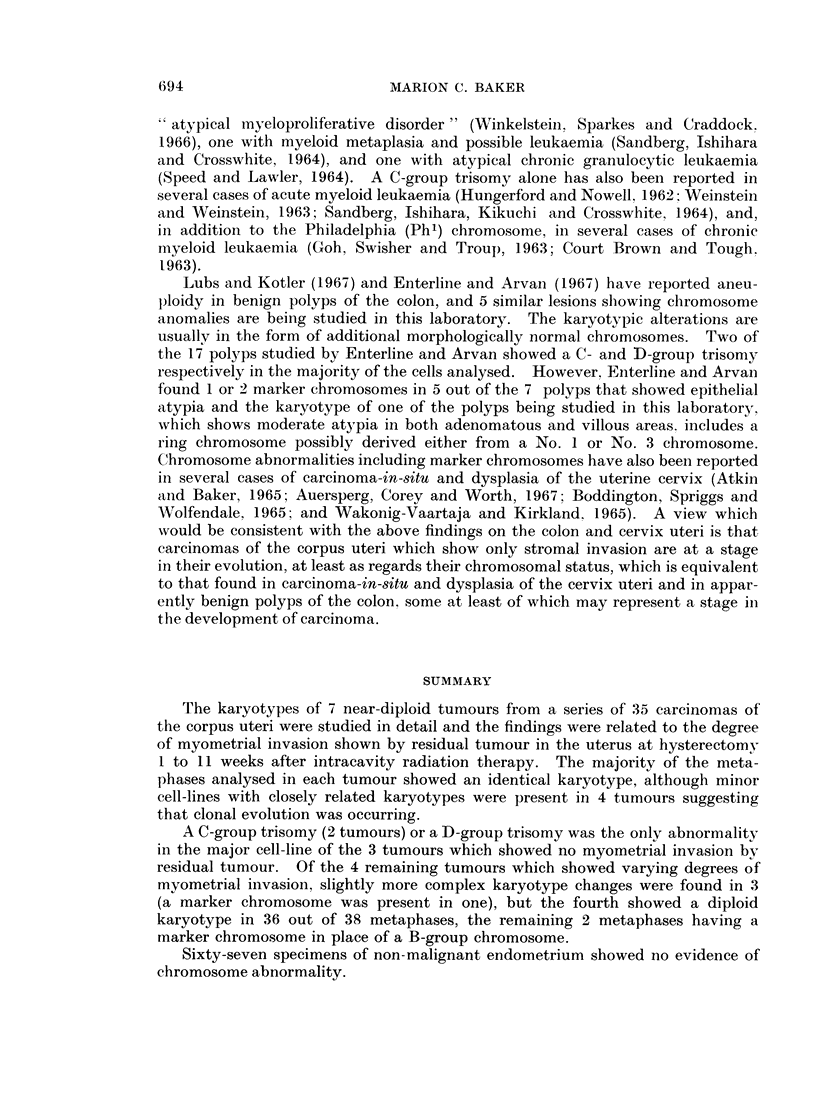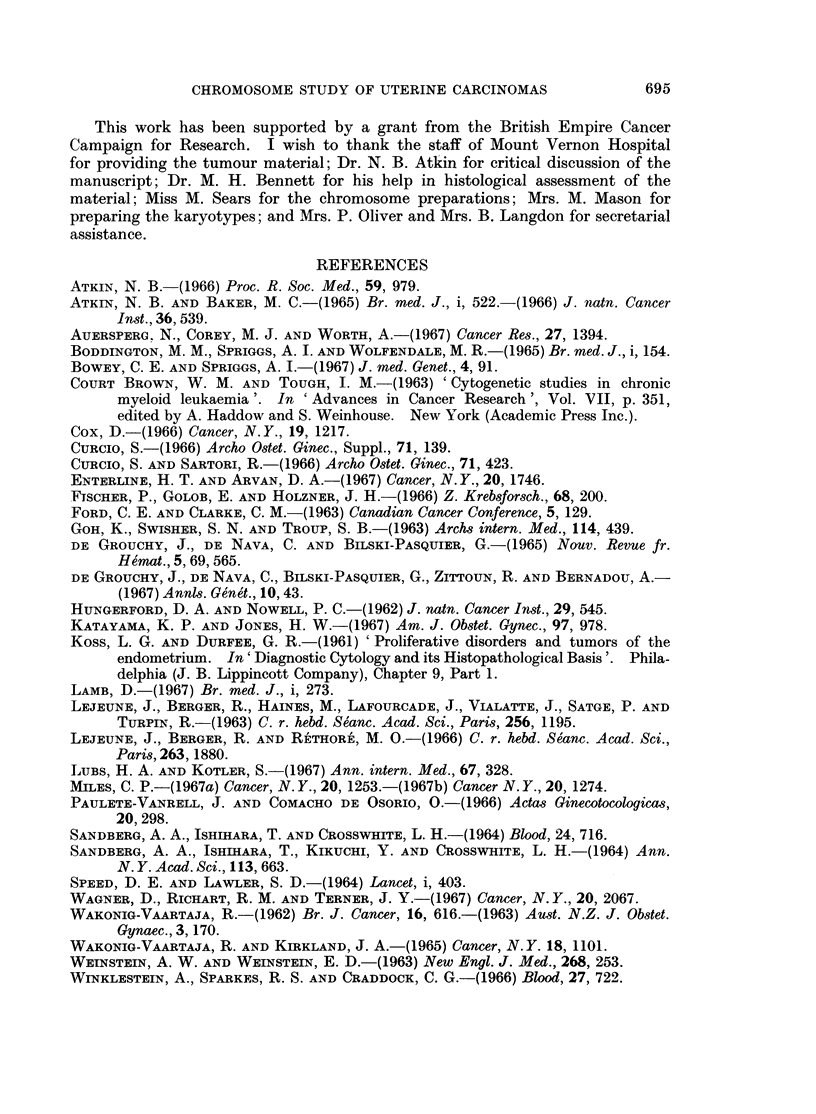# A chromosome study of seven near-diploid carcinomas of the corpus uteri.

**DOI:** 10.1038/bjc.1968.81

**Published:** 1968-12

**Authors:** M. C. Baker

## Abstract

**Images:**


### A CHROMOSOME STUDY OF SEVEN NEAR-DIPLOID CARCINOMAS OF THE CORPUS UTERI

MARION C. BAKER

*From the Department of Cancer Research, Mount Vernon Hospital, Northwood, Middlesex*

Received for publication July 4, 1968

CHROMOSOME studies on invasive tumours have shown that in general each possesses a unique abnormal karyotype. There is little information at present on the course of development of malignant cells with abnormal karyotypes from diploid cells. It is perhaps from those lesions described as carcinoma-*in-situ* and other lesions which may have a malignant potential (including some histologically benign or " atypical " polyps and hyperplasias), as well as from those malignant lesions which show the earliest invasive changes, that information relating to the importance of chromosomes in the malignant transformation might be obtained.

A comparative study is therefore in progress of normal endometrium, endometrium showing benign pathological conditions and carcinoma of the endometrium. This report describes the chromosome findings on 7 cases of carcinoma of the corpus uteri having modal chromosome numbers in the range of 46–49, and preliminary results on 67 specimens of non-malignant endometrium. Tumours in the near-diploid range were chosen for study since these might be expected to show least deviation from the normal karyotype, and any common pattern might be most clearly revealed. DNA measurements suggest that most carcinomas of the corpus uteri in fact fall in the diploid range (Atkin, 1966). The tumours were selected on the basis of quality of chromosome preparations from 31 near-diploid tumours which form the majority of a series of 35 carcinomas of the corpus uteri (of the remaining 4, 2 showed both near-diploid and near-tetraploid modes and 2 showed near-triploid modes).

Observations were made on the untreated specimens; all the patients received from 1–3 Stockholm radium or Cobalt-60 insertions followed after an interval of 1–11 weeks by hysterectomy. Particular attention was paid to the depth of myometrial invasion shown by any residual tumour seen in the hysterectomy specimen.

### MATERIALS AND METHODS

Tumour material obtained by endometrial curettage was divided as follows:

(1) Part was fixed in 10% formol saline for routine histological study.

(2) A small piece was fixed in acetic alcohol; from this, an orcein squash preparation was made and examined for the presence of tumour, and especially for the frequency of tumour metaphases. Sex chromatin was evaluated in tumour interphase cells on a second well-flattened orcein squash preparation.

(3) The remainder was pretreated for chromosome studies by a direct method similar to that previously described (Atkin and Baker, 1966). The less well differentiated tumours have been found to yield more satisfactory results when exposed to hypotonic solution for only 15 minutes.

The uteri removed after radiation therapy were examined macroscopically, and areas showing possible residual tumour were chosen for histological examination. When no tumour was apparent, samples from each cornu and the anterior and posterior wall were examined histologically for tumour, including evidence of radiation-destroyed tumour in the form of non-viable tumour giant cells.

To exclude any possible constitutional chromosome anomaly, the karyotypes of normal leucocytes from each patient were studied in cultures derived from either an uninvaded pelvic lymph node removed at hysterectomy or peripheral blood.

Curettings from non-malignant cases were pretreated for chromosome studies by a similar direct technique, provided that preliminary examination of an orcein squash preparation showed a sufficient number of mitoses in the epithelium.

### RESULTS

### *Case* 1

Aged 52. Endometrial curettings showed a moderately well-differentiated adenocarcinoma. Treatment consisted of 2 Cobalt-60 insertions with a week's interval in between, followed 7 weeks later by panhysterectomy. Histological examination showed considerable radiation changes in the uterus and ovaries but no evidence of neoplastic growth in the uterus, tubes, ovaries or lymph nodes. The patient was well 15 months later.

Chromosome counts on the untreated specimen were as follows:—

| | Chromosome number | | | | | | |
|---|---|---|---|---|---|---|---|
| | 42 | 43 | 44 | 45 | 46 | 47 | Total |
| No. of cells | 1 | 1 | 1 | 5 | 12 | 21 | 41 |

Sixteen metaphases with 47 chromosomes were analysed and all showed an apparent trisomy for a C-group chromosome (Fig. 1). In 2 cells a normal diploid karyotype was present. Four hypodiploid cells were analysed: 3 metaphases with 45 chromosomes showed loss of a C-, D- and E-group chromosome respectively, and 1 metaphase with 44 chromosomes lacked a C- and an E-group chromosome.

### *Case* 2

Aged 51. Endometrial curettings showed atypical hyperplastic endometrium with areas of frank well-differentiated adenocarcinoma. The patient was treated by 1 Stockholm radium insertion followed 3 weeks later by an extended hysterectomy. Histologically the uterus showed a few areas of grossly atypical endometrial glands but no invasive carcinoma. The patient was well 20 months later.

Chromosome counts on the untreated specimen showed:

| | Chromosome number | | | | | |
|---|---|---|---|---|---|---|
| | 38 | 41 | 45 | 46 | 47 | Total |
| No. of cells | 1 | 1 | 1 | 7 | 22 | 32 |

Karyotypes of 13 metaphases with 47 chromosomes showed, as in the previous case, an apparent trisomy for a C-group chromosome (Fig. 2). This abnormality was present in 2 cells with 46 chromosomes which also lacked an E-group chromosome. Two further metaphases with 46 chromosomes showed normal diploid karyotypes. Thus 15 of a total of 17 cells analysed carried the apparent C-group trisomy.

*Case* 3

Aged 53. Endometrial curettage was performed on 2 occasions (at a week's interval) before treatment, but only tissue from the second specimen was used for chromosome study. Histologically the first specimen showed a well-differentiated papillary adenocarcinoma. On the second occasion, both the tissue fixed in formol saline and a small piece of the material taken for chromosome studies which was subsequently examined histologically showed only atypical adenomatous hyperplasia. Treatment consisted of 2 Cobalt-60 insertions at a week's interval, followed 11 weeks later by panhysterectomy. Histologically, the uterus showed a small superficial area of papillary adenocarcinoma, with no invasion of the myometrium. The patient was well 21 months later.

Chromosome counts on the untreated specimen showed:—

| | Chromosome number | | | | | | | | | |
|---|---|---|---|---|---|---|---|---|---|---|
| | 42 | 43 | 44 | 45 | 46 | 47 | 48 | 50 | 51 | Total |
| No. of cells | 4 | 1 | 5 | 7 | 14 | 41 | 12 | 5 | 3 | 92 |

A major cell-line with an apparent D-group trisomy was revealed in 33 out of 35 cells analysed with 47 chromosomes (Fig. 3). Three minor cell-lines with 48 (Fig. 4), 50 and 51 chromosomes show closely related karyotypes which include the apparent D-group trisomy (Table I).

In addition to those summarised in Table I, 7 cells with either 44 or 45 chromosomes were analysed: 5 showed the apparent D-group trisomy; otherwise there was random chromosome loss from groups C, E, F and G. The metaphases with 46 or 47 chromosomes which differ from the 47 or 48 chromosome cell-lines by the random loss of one chromosome could represent minor cell-lines, but it seems more probable that they are members of the 47 and 48 chromosome cell-lines respectively and have suffered chromosome loss.

*Case* 4

Aged 58. Two specimens of endometrial curettings were obtained at an interval of a week before treatment and both showed a well-differentiated adenoacanthoma. Clinically the tumour was close to the endocervix but nevertheless appeared confined to the corpus.

Treatment consisted of 3 Stockholm radium insertions with intervals of 1 and 2 weeks respectively, followed 3 weeks later by Wertheim's hysterectomy. Histologically the uterus showed residual viable adenoacanthoma just commencing invasion on the posterior endometrial surface. No metastases were found. Thirty months after initial diagnosis, an emergency resection of a portion of small intestine for obstruction revealed a malignant stricture which histologically showed moderately differentiated adenocarcinoma similar to that seen previously in the corpus uteri.

Chromosome studies were performed on both the untreated specimens; the chromosome counts are shown below.

| | | Chromosome number | | | | | | | |
|---|---|---|---|---|---|---|---|---|---|
| | | <44 | 44 | 45 | 46 | 47 | 48 | 94 | Total |
| No. of cells | 1st Specimen | 6 | 2 | 2 | 13 | 62 | 11 | — | 96 |
| | 2nd Specimen | 2 | 3 | 3 | 4 | 19 | 4 | 1 | 36 |
| | Total | 8 | 5 | 5 | 17 | 81 | 15 | 1 | 132 |

Analysis of metaphases from each specimen showed no significant difference and the findings have therefore been pooled in Table I.

At least 3 related cell-lines having 46, 47 and 48 chromosomes respectively appear to be present in this tumour. The major cell-line has 47 chromosomes; its karyotype contains a submetacentric marker chromosome longer than the No. 1 chromosomes, other changes being the addition of a C- and an F-group chromosome and the loss of a No. 2 and a No. 16 chromosome (Fig. 5). Most of the cells with 46 chromosomes showed a similar karyotype including the marker chromosome but without the additional F-group chromosome (Fig. 6), and they probably represent a distinct but related cell-line. The same may be true of the 3 cells with 48 chromosomes which had the same karyotype as the 47 chromosome cell-line with a second additional C-group chromosome.

In addition to those summarised in Table I, 2 metaphases with less than 46 chromosomes which may have suffered chromosome loss were analysed. A cell with 45 chromosomes showed the submetacentric marker chromosome but had lost a No. 2 and No. 16 chromosome. A metaphase with 44 chromosomes differed from the 47 chromosome cell-line in lacking a No. 3, a second E-group and a G-group chromosome. The cells with 46 or 47 chromosomes whose karyotypes differ from that of the majority with the same chromosome numbers might represent further minor cell-lines, but it seems more probable that they are incomplete cells, analysis showing that they lacked one chromosome as compared with one or other of the 3 distinct cell-lines.

Comparative measurements of the long marker chromosome and the single apparently normal No. 2 chromosome in 10 metaphases from the 47 chromosome cell-line showed that the mean arm ratio of the markers was 1·96 (standard deviation $\pm$ 0·22) while that of the apparently normal No. 2 chromosomes was 1·56 (standard deviation $\pm$ 0·17); the mean length of the markers relative to that of the No. 2 chromosomes was 1·24 (standard deviation $\pm$ 0·08). In comparison, the mean arm ratios of the two No. 2 chromosomes in 8 diploid metaphases from the series of non-malignant endometria (estimating the average ratio for the chromosome in each cell with the longer arm ratio, and that for the chromosome with the shorter arm ratio) was 1·65 (standard deviation $\pm$ 0·18) and 1·56 (standard deviation $\pm$ 0·13) respectively; the mean ratio of the lengths of these No. 2 chromosomes (longer chromosome/shorter chromosome) was 1·04 (standard deviation $\pm$ 0·06). The No. 2 chromosomes in 3 diploid metaphases from this patient's leucocyte culture had the following arm ratios (the first figure refers to the longer chromosome, and the ratio of the length of the longer to that of the shorter chromosome is given in brackets after each pair): 1·70 and 1·60 (1·04); 1·46 and 1·58 (1·03); 1·45 and 1·45 (1·00).

*Case* 5

Aged 67. Endometrial curettings showed a moderately well-differentiated columnar cell adenocarcinoma. An extended hysterectomy was performed 1 week after a Stockholm radium insertion. Tumour of similar histological appearance was present on the posterior wall of the uterus, which it had invaded to a depth of 7 mm.; tumour was also extending down the cervical canal. No tumour was present in the parametria, ovaries or pelvic lymph nodes. The patient was well 10 months later.

Chromosome counts on the untreated specimen were as follows:

| | Chromosome number | | | | | | | |
|---|---|---|---|---|---|---|---|---|
| | 44 | 45 | 46 | 47 | 48 | 49 | 51 | Total |
| No. of cells | 2 | 1 | 2 | 5 | 7 | 33 | 1 | 51 |

A major cell-line with 49 chromosomes whose karyotype shows 3 additional C-group chromosomes was present in this tumour (Fig. 7). The metaphases with less than 49 chromosomes which were analysed are probably incomplete although a minor cell-line with 48 chromosomes showing only 2 additional C-group chromosomes may be present. There may also be a second minor cell-line with 51 chromosomes (Table I).

*Case* 6

Aged 73.—Endometrial curettings showed a well-differentiated papillary adenocarcinoma. Treatment consisted of 2 Stockholm radium insertions with an interval of a week followed 4 weeks later by Wertheim's hysterectomy. Histologically, the uterus showed tumour extending to within 6 mm. of the serosal surface, but no metastases were found. The patient was well 26 months later.

Chromosome counts obtained from the untreated specimen were as follows:

| | Chromosome number | | | | | | |
|---|---|---|---|---|---|---|---|
| | <40 | 40 | 42 | 43 | 45 | 46 | Total |
| No. of cells | 5 | 2 | 1 | 1 | 9 | 34 | 52 |

Of 23 metaphases analysed with 46 chromosomes, 1 showed an apparently normal diploid karyotype. The remaining 22 revealed an additional G-group chromosome and the loss of a No. 16 chromosome (Fig. 8). Five cells with 45 chromosomes showed the same abnormal karyotype less either a D-, a second E- or an F-group chromosome. A further cell with 45 chromosomes showed only the loss of a No. 16 chromosome from a diploid karyotype. Thus, of 29 cells analysed, 27 showed the additional G-group chromosome.

TABLE I.—*Seven Near-Diploid Tumours of the Corpus Uteri: Histology, Treatment and Chromosomal Findings*

| Case Number | Histology immediately prior to treatment | Number of Stockholm radium or Cobalt-60 insertions | Interval between beginning of radiotherapy and hysterectomy | Hysterectomy findings | Chromosomal findings on untreated tumours: Number of chromosomes | Number of cells analysed | Karyotype |
|---|---|---|---|---|---|---|---|
| 1. | Moderately well differentiated adenocarcinoma | 2 Cobalt-60 | 8 weeks | No tumour present | 47 | 16 | N+1C |
| | | | | | 46 | 2 | N |
| 2. | Atypical hyperplastic endometrium with areas of frank well-differentiated adenocarcinoma | 1 radium | 3 weeks | Few areas of residual grossly atypical glands—no invasive carcinoma | 47 | 13 | N+1C |
| | | | | | 46 | 2 | N+1C−1E |
| | | | | | 46 | 2 | N |
| 3. | Atypical adenomatous hyperplasia (curettings a week previously: well-differentiated papillary adenocarcinoma) | 2 Cobalt-60 | 12 weeks | Small superficial area of papillary adenocarcinoma—no myometrial infiltration | 51 | 2 | N+2D+2C+1E |
| | | | | | 50 | 4 | N+2D+2C |
| | | | | | 48 | 3 | N+1D+1C |
| | | | | | 47 | 1 | N+1D+1C −1G |
| | | | | | 47 | 1 | N +1C |
| | | | | | 47 | 33 | N+1D |
| | | | | | 46 | 3 | N |
| | | | | | 46 | 1 | N+1D −1E |
| | | | | | 46 | 1 | N+1D −1F |
| | | | | | 46 | 2 | N+1D −1G |
| 4. | Well-differentiated adenoacanthoma | 3 radium | 6 weeks | Residual viable superficial adenoacanthoma, just commencing invasion | 48 | 3 | N+1smM−1A$_2$+2C+1F−IE$_{16}$ |
| | | | | | 47 | 1 | N+1smM−1A$_2$+2C −1E$_{16}$ |
| | | | | | 47 | 36 | N+1smM−1A$_2$+1C+1F−1E$_{16}$ |
| | | | | | 46 | 6 | N+1smM−1A$_2$+1C −1E$_{16}$ |
| | | | | | 46 | 1 | N+1smM−1A$_2$ +1F−1E$_{16}$ |
| | | | | | 46 | 1 | N+1smM−1A$_2$+1C+1F−IE$_{16}$−1G |
| 5. | Moderately well-differentiated columnar cell adenocarcinoma | 1 radium | 1 week | Tumour on posterior wall of corpus uteri extending into the wall to a depth of 7 mm. and down the endocervical canal | 51 | 1 | N+4C+1G |
| | | | | | 49 | 24 | N+3C |
| | | | | | 48 | 1 | N+3C−1B |
| | | | | | 48 | 1 | N+3C−1D |
| | | | | | 48 | 2 | N+2C |
| | | | | | 47 | 1 | N+2C−1F |
| 6. | Well-differentiated papillary adenocarcinoma | 2 radium | 5 weeks | Tumour extending to within 6 mm. of serosal surface | 46 | 22 | N+1G−1E$_{16}$ |
| | | | | | 46 | 1 | N |
| 7. | Undifferentiated adenocarcinoma | 1 radium | 2 weeks | Tumour extending through myometrium to serosal surface, and secondary deposits in left ovary and peritoneum | 46 | 2 | N+1stM−1B |
| | | | | | 46 | 36 | N |

N = normal diploid complement; the additional or missing chromosomes are indicated; smM = submetacentric marker; stM = subtelocentric marker.

*Case* 7

Aged 57. Endometrial curettings showed an undifferentiated adenocarcinoma. Treatment consisted of a Stockholm radium insertion followed after 2 weeks by total hysterectomy. Histologically, tumour was found to be extending from the uterine cavity through the myometrium to the serosal surface, and the deposits were present in the left ovary and peritoneum. Ten days later, firm discrete nodes were felt in both inguinal regions which were considered to contain metastatic tumour. The patient was given 2 courses of external radiation but died 9 months after initial diagnosis.

Chromosome counts from the untreated specimen showed:

| | Chromosome number | | | | | | | |
|---|---|---|---|---|---|---|---|---|
| | <40 | 40 | 42 | 43 | 44 | 45 | 46 | Total |
| No. of cells | 3 | 2 | 1 | 5 | 2 | 7 | 57 | 77 |

Thirty-eight metaphases with 46 chromosomes were analysed. Thirty-six of these showed an apparently normal diploid karyotype (Fig. 9) but in 2 cells which perhaps represent a minor cell-line a subtelocentric marker chromosome larger than the No. 1 chromosome replaced a B-group chromosome (Fig. 10). Two cells with 45 chromosomes were analysed and showed the loss of a C-group and an E-group chromosome respectively from a diploid karyotype.

Careful scrutiny of the analysed metaphases for any small but consistent chromosomal changes revealed only a possibly abnormally large No. 17 chromosome pair in 15 of the 38 cells with 46 chromosomes, including the 2 with the marker chromosome (Fig. 10). It is possible that the apparently normal metaphases originated from non-malignant epithelium adjacent to the tumour or from the stroma. However, the tissue used for chromosome studies was homogeneous in appearance and neither the orcein squash preparation nor the histological sections showed the presence of any non-malignant endometrium. Very few mitoses were present in the stroma.

An orcein squash preparation of tumour material from the hysterectomy specimen revealed many mitoses a large number of which showed evidence of radiation damage but a chromosome preparation was of poor quality. Specimens of metastatic tumour obtained 24 hours *post mortem* showed many mitoses but no countable metaphases were obtained from a chromosome preparation.

A brief report of this case was published previously (Atkin and Baker, 1966).

A single sex chromatin body was present in the interphase cells of all these tumours. The leucocyte cultures yielded only diploid karyotypes.

Of the 24 near-diploid tumours which yielded less favourable chromosome preparations, 13 have so far been assessed for the presence of marker chromosomes; 6 of these tumours showed at least 1 marker chromosome. These 13 tumours showed a moderately or well-differentiated histological pattern, apart from 1 tumour (having a marker chromosome) which was poorly differentiated. Of the 11 tumours in which the presence of a marker chromosome has not yet been assessed, only 1 was poorly differentiated. Both near-diploid and near-tetraploid modes were present in 2 further adenocarcinomas. One of these was poorly differentiated and the other well-differentiated, but only the former showed a marker chromosome. The karyotypes of a well-differentiated and a poorly differentiated adenocarcinoma with chromosome numbers in the range of 60–80 included a marker chromosome. No correlation is so far apparent between the modal chromosome numbers of these 28 tumours and the depth of myometrial invasion of residual tumour present in the hysterectomy specimens.

Analysable metaphases were obtained from 67 specimens of *non-malignant endometrium*; 47 of these were in the proliferative phase and 20 in the secretory. Eighteen of the former showed hyperplastic changes, 3 being classified as metropathia. Polypoid changes were present in 7 of the 67 cases and areas of atypical epithelium were seen in 1 case included in the hyperplastic group and in 1 included in the proliferative group. The analyses are incomplete but none has yielded chromosome counts greater than 46, although 36 out of the 106 metaphases analysed so far have counts of between 36 and 45. This hypodiploidy could in each instance have arisen by the loss of 1 or more chromosomes from a diploid karyotype. The frequency of hypodiploid metaphases shows no correlation with the histological pattern of the endometrium.

### DISCUSSION

The 7 cases of carcinoma of the corpus uteri presented in this study provide further examples of the diversity of karyotype to be found even among a group of tumours from the same site and with similar chromosome numbers.

Both Cases 1 and 2 showed an apparent C-group trisomy and may therefore have undergone an identical chromosome change; however, it is possible that the extra chromosome is different in the 2 tumours and, in either or both, could be an abnormal chromosome, perhaps derived from chromosomes of groups other than C-group.

A further important finding in all 7 cases is the presence of an identical karyotype in the majority of the metaphases from each tumour. This supports the hypothesis that all the cells within a tumour commonly arise from 1 cell with an abnormal karyotype.

---

### EXPLANATION OF PLATES.

FIG. 1.—Case No. 1.—Karyotype of metaphase with 47 chromosomes showing a C-group trisomy.

FIG. 2.—Case No. 2.—Karyotype of metaphase with 47 chromosomes showing a C-group trisomy.

FIG. 3.—Case No. 3.—Karyotype of metaphase from the major cell-line with 47 chromosomes showing a D-group trisomy.

FIG. 4.—Case No. 3.—Karyotype of metaphase with 48 chromosomes showing a C- and D-group trisomy.

FIG. 5.—Case No. 4.—Karyotype of metaphase from the major cell-line with 47 chromosomes showing an additional C- and F-group chromosome and the loss of a No. 16 chromosome. The marker chromosome (arrowed) replaces a No. 2 chromosome.

FIG. 6.—Case No. 4.—Karyotype of metaphase with 46 chromosomes showing an additional C-group chromosome and the loss of a No. 16 chromosome. The marker chromosome (arrowed) replaces a No. 2 chromosome.

FIG. 7.—Case No. 5.—Karyotype of metaphase with 49 chromosomes showing 3 additional C-group metaphases.

FIG. 8.—Case No. 6.—Karyotype of metaphase with 46 chromosomes showing the loss of a No. 16 chromosome and the addition of a G-group chromosome.

FIG. 9.—Case No. 7.—Karyotype of metaphase with 46 chromosomes showing an apparently diploid karyotype.

FIG. 10.—Case No. 7.—Karyotype of metaphase with 46 chromosomes showing the marker chromosome (arrowed) replacing a B-group chromosome and including the possibly abnormally large No. 17 chromosome pair.

1

2

Baker.

3

Baker.

4

5

Baker.

6

8

Baker.

9

10

Baker.

A few cells with differing but closely related karyotypes have been found in each tumour. Some of these may represent divergent cell-lines but others may have resulted from chromosome loss due to cell breakage. The metaphases from Cases 1, 2, 3 and 6 which show a diploid karyotype may have originated from adjacent non-malignant epithelium or from the stroma. However, in Cases 1, 2 and 3 whose tumour cell-lines showed only additional morphologically normal chromosomes to an otherwise diploid karyotype the apparently normal metaphases could be incomplete tumour cells.

Several workers have formulated the clonal evolution theory according to which the malignant cell population develops through a number of stages involving chromosomal changes (Ford and Clarke, 1963; Lejeune, Berger, Haines, Lafourcade, Vialatte, Satge and Turpin, 1963; and de Grouchy, de Nava and Bilski-Pasquier, 1965). The divergent cell-lines in Cases 3, 4, 5 and 7 may represent new clones derived from the major cell-line, or alternatively earlier clones from which the major cell-line has evolved. In Cases 1, 2 and 6 less than 30 cells were analysed, perhaps too few to reveal the presence of any variant cell-lines.

In Case 3, most of the metaphases showed only an apparent D-group trisomy, and the cells with 48, 50 and 51 chromosomes may have been derived from this line by the sequential addition of 1 or 2 chromosomes. Lejeune *et al.* (1963) described in a trisomic mongol the acquisition by the leukaemia cells of a supernumary chromosome which was then duplicated; this was followed by the acquisition of further additional chromosomes which were in turn duplicated, thus giving rise to the major 54-chromosome cell line. de Grouchy, de Nava and Bilski-Pasquier (1965) have found evidence of a similar stepwise evolutionary pattern in 2 cases of chronic myeloid leukaemia. It is possible that a similar process of clonal evolution was occurring in the tumour of Case 3 at the time of presentation for treatment.

In Case 4 the 46- and 48-chromosome cell-lines differed by 1 chromosome from the major cell-line with 47 chromosomes. The 47-chromosome cell-line might have developed either from the 46–chromosome cell-line or directly from a diploid cell; in the latter case, the 46-chromosome cell-line may have arisen from the 47-chromosome cell-line by the loss of an F-group chromosome. The 48-chromosome cell-line probably evolved directly from the 47-chromosome cell-line by the addition of a C-group chromosome.

In Case 5 a minor cell-line with 48 chromosomes may have been present from which the major cell-line with 49 chromosomes could have evolved by the addition of a C-group chromosome. The 1 cell with 51 chromosomes may represent a further step in the evolution of this tumour.

It is probable that non-disjunction led to the acquisition of the additional morphologically normal chromosomes present in the karyotypes of Cases 1–5, but endoreduplication could also have been responsible. Evidence for endoreduplication of a single chromosome in metaphases derived from normal fibroblast cultures (Lejeune, Berger and Rethoré, 1966) and from a 48-hour marrow culture from a case of multiple myeloma (de Grouchy, de Nava, Bilski-Pasquier, Zittoun and Bernadou 1967) has been presented.

In the abnormal karyotype of Case 6 there appear to be an extra G-group chromosome and a missing No. 16 chromosome, but one of the chromosomes placed in G-group could be the missing No. 16 chromosome which has suffered a deletion. In one of a series of 7 nephroblastomas, Cox (1966) found a pseudodiploid karyotype, a No. 1 chromosome being apparently replaced by a C-group chromosome; he suggested that the latter was in fact a No. 1 chromosome which had suffered a deletion. Structural changes have also evidently occurred in the evolution of the abnormal karyotype present in Case 4, and in the 2 cells of Case 7 which show a marker chromosome and perhaps represent a developing clone.

The majority of the cells in Case 7 had an apparently normal diploid chromosome complement, although it is possible that a chromosome change had occurred which did not alter the final appearance of the karyotype, such as the substitution of a chromosome by another of similar appearance or a deletion or duplication too small to be detectable. Reports of apparently normal karyotypes in uncultured material from malignant tumours are few. Cox (1966) has described 3 nephroblastomas with diploid karyotypes. Miles (1967*a*) has reported a similar finding in a neuroblastoma, and in a lymph node metastasis from a carcinoma of the breast (Miles, 1967*b*). A nephroblastoma under investigation in this laboratory also appears to have a diploid karyotype. Curcio (1966), studying a granulosa-cell tumour of the ovary, found only diploid karyotypes in the tumour cells analysed from the primary lesion, omental metastases and ascitic fluid.

In the present series, a marker chromosome was only found in Case 4 and in 2 cells from Case 7. Wakonig-Vaartaja (1962) described a well-differentiated adenocarcinoma of the corpus uteri with a modal chromosome number of 46 whose karyotype included a submetacentric marker chromosome approximately twice as long as the single No. 1 chromosome. In a further series of carcinomas of the corpus uteri, Wakonig-Vaartaja (1963) described 4 cases with near-diploid chromosome numbers. Variable chromosomal alterations, including a few marker chromosomes, were present in the first case, a well-differentiated adenocarcinoma. One cell was analysed from a second case, a moderately-differentiated adenocarcinoma; it had 50 chromosomes and its karyotype showed only an additional No. 2 chromosome and 3 additional C-group chromosomes. A third case, a well-differentiated adenocarcinoma showed a modal chromosome number of 46; one metaphase analysed revealed a diploid karyotype, but it was considered possibly to be a non-malignant cell.

Recently, Katayama and Jones (1967), in a study of 5 carcinomas of the corpus uteri, reported the presence of 2 marker chromosomes, the first being similar to a B-group chromosome with a deleted short arm and the second a centric fragment, in a grade 4 anaplastic adenocarcinoma with chromosome numbers in the range of 55–61. A second case, a grade 1 well-differentiated adenocarcinoma with chromosome numbers in the range of 47–52, showed only additional C- and E-group chromosomes. No karyotype details were given of the other 3 cases in this series; 2 of these, a grade 2 adenoacanthoma and a grade 4 adenosquamous carcinoma, had hyperdiploid chromosome numbers, whilst the third case, an adenocarcinoma grade 3 showed chromosome numbers varying from 47 to 68.

Fischer, Golob and Holzner (1966) have described 2 adenocarcinomas of the corpus uteri with modal chromosome numbers of 32 and 59 respectively. Only 1 karyotype (from the latter tumour) was depicted; it showed 3 marker chromosomes. Curcio and Sartori (1966) have described 2 carcinomas of the corpus uteri with hypodiploid and hypotriploid modal chromosome numbers respectively, but no details of the karyotypes were given. An endometrial carcinoma with a modal chromosome number of 74, with marker chromosomes, was described by Paulete-Vanrell and Comacho de Osorio (1966).

Preliminary data on the remaining 28 tumours of the corpus uteri from the series of 35 being studied in this laboratory, most of which yielded less satisfactory chromosome preparations, have shown marker chromosomes of variable morphology to be present in some, and suggest that marker chromosomes may be more frequently found in poorly-differentiated tumours and in those with higher chromosome numbers; this is also supported by the findings of the other workers described above.

Lamb (1967), in a series of transitional-cell carcinomas of the bladder, found that near-diploid chromosome counts predominated in tumours with a well-differentiated histological pattern but the majority of these were classified as *in-situ* carcinomas. Most of the invasive tumours were either moderately well-differentiated with near-tetraploid modal chromosome numbers or undifferentiated with near-triploid modes. No correlation was evident among the invasive tumours between modal chromosome number and depth of invasion; a similar lack of correlation has so far been found in the present series of carcinomas of the corpus uteri.

No relationship is apparent between the karyotypes of the 7 tumours presented in detail in this study and their degree of differentiation. It may however be significant that the hysterectomy specimens of Cases 1 to 3, which were the only ones which showed a *single* additional morphologically normal chromosome in their major cell-lines, revealed no histological evidence of myometrial invasion by residual tumour. It has been suggested that any endometrial carcinoma confined to the endometrium should be regarded as carcinoma-*in-situ* (Koss and Durfee, 1961). Such a description would apply to Cases 1 to 3. The curettings obtained before treatment from Case 2 showed areas of atypical hyperplasia as well as adenocarcinoma, and the metaphases that were analysed could have originated from either of these. A similar possibility exists for Case 3: the tissue used for chromosome studies showed only atypical adenomatous hyperplasia but previous curettings and the subsequent hysterectomy specimen both showed adenocarcinoma. However, in the present series of 67 non-malignant endometria, which includes 2 cases with atypical epithelial changes, 3 of metropathia and 18 of hyperplasia, no chromosome abnormalities have so far been found. Bowey and Spriggs (1967) in a study of non-malignant endometria also found only normal karyotypes apart from hypodiploidy due to inconsistent chromosome loss. Wakonig-Vaartaja (1963) in a study of 16 similar cases found normal karyotypes in the majority of the cells analysed and considered the variations to have arisen during preparation. Curcio and Sartori (1966) have studied 2 cases of endometrial hyperplasia, but only 9 cells, from 1 of the cases, were analysed; all were diploid.

Wagner, Richart and Terner (1967) have reported finding a wide range of high interphase DNA values in some endometria showing only glandular hyperplasia. It is difficult to relate these results on the one hand to the absence of chromosome abnormalities in non-malignant endometrium and on the other to the minimal changes (in most metaphases, trisomy for a single chromosome) found in the 3 tumours in the present series showing only stromal invasion.

Several malignant or premalignant lesions in which trisomies were the only chromosomal alterations have been reported. A D–group trisomy was present in the myeloma cells of the case mentioned above showing possible selective endoreduplication (de Grouchy *et al.*, 1967). C-group trisomy has been reported as the only chromosomal abnormality in the bone marrow of a patient with

" atypical myeloproliferative disorder " (Winkelstein, Sparkes and Craddock, 1966), one with myeloid metaplasia and possible leukaemia (Sandberg, Ishihara and Crosswhite, 1964), and one with atypical chronic granulocytic leukaemia (Speed and Lawler, 1964). A C-group trisomy alone has also been reported in several cases of acute myeloid leukaemia (Hungerford and Nowell, 1962; Weinstein and Weinstein, 1963; Sandberg, Ishihara, Kikuchi and Crosswhite, 1964), and, in addition to the Philadelphia ($Ph^1$) chromosome, in several cases of chronic myeloid leukaemia (Goh, Swisher and Troup, 1963; Court Brown and Tough, 1963).

Lubs and Kotler (1967) and Enterline and Arvan (1967) have reported aneuploidy in benign polyps of the colon, and 5 similar lesions showing chromosome anomalies are being studied in this laboratory. The karyotypic alterations are usually in the form of additional morphologically normal chromosomes. Two of the 17 polyps studied by Enterline and Arvan showed a C- and D-group trisomy respectively in the majority of the cells analysed. However, Enterline and Arvan found 1 or 2 marker chromosomes in 5 out of the 7 polyps that showed epithelial atypia and the karyotype of one of the polyps being studied in this laboratory, which shows moderate atypia in both adenomatous and villous areas, includes a ring chromosome possibly derived either from a No. 1 or No. 3 chromosome. Chromosome abnormalities including marker chromosomes have also been reported in several cases of carcinoma-*in-situ* and dysplasia of the uterine cervix (Atkin and Baker, 1965; Auersperg, Corey and Worth, 1967; Boddington, Spriggs and Wolfendale, 1965; and Wakonig-Vaartaja and Kirkland, 1965). A view which would be consistent with the above findings on the colon and cervix uteri is that carcinomas of the corpus uteri which show only stromal invasion are at a stage in their evolution, at least as regards their chromosomal status, which is equivalent to that found in carcinoma-*in-situ* and dysplasia of the cervix uteri and in apparently benign polyps of the colon, some at least of which may represent a stage in the development of carcinoma.

### SUMMARY

The karyotypes of 7 near-diploid tumours from a series of 35 carcinomas of the corpus uteri were studied in detail and the findings were related to the degree of myometrial invasion shown by residual tumour in the uterus at hysterectomy 1 to 11 weeks after intracavity radiation therapy. The majority of the metaphases analysed in each tumour showed an identical karyotype, although minor cell-lines with closely related karyotypes were present in 4 tumours suggesting that clonal evolution was occurring.

A C-group trisomy (2 tumours) or a D-group trisomy was the only abnormality in the major cell-line of the 3 tumours which showed no myometrial invasion by residual tumour. Of the 4 remaining tumours which showed varying degrees of myometrial invasion, slightly more complex karyotype changes were found in 3 (a marker chromosome was present in one), but the fourth showed a diploid karyotype in 36 out of 38 metaphases, the remaining 2 metaphases having a marker chromosome in place of a B-group chromosome.

Sixty-seven specimens of non-malignant endometrium showed no evidence of chromosome abnormality.

This work has been supported by a grant from the British Empire Cancer Campaign for Research. I wish to thank the staff of Mount Vernon Hospital for providing the tumour material; Dr. N. B. Atkin for critical discussion of the manuscript; Dr. M. H. Bennett for his help in histological assessment of the material; Miss M. Sears for the chromosome preparations; Mrs. M. Mason for preparing the karyotypes; and Mrs. P. Oliver and Mrs. B. Langdon for secretarial assistance.

### REFERENCES

ATKIN, N. B.—(1966) *Proc. R. Soc. Med.*, **59**, 979.
ATKIN, N. B. AND BAKER, M. C.—(1965) *Br. med. J.*, i, 522.—(1966) *J. natn. Cancer Inst.*, **36**, 539.
AUERSPERG, N., COREY, M. J. AND WORTH, A.—(1967) *Cancer Res.*, **27**, 1394.
BODDINGTON, M. M., SPRIGGS, A. I. AND WOLFENDALE, M. R.—(1965) *Br. med. J.*, i, 154.
BOWEY, C. E. AND SPRIGGS, A. I.—(1967) *J. med. Genet.*, **4**, 91.
COURT BROWN, W. M. AND TOUGH, I. M.—(1963) 'Cytogenetic studies in chronic myeloid leukaemia'. *In* 'Advances in Cancer Research', Vol. VII, p. 351, edited by A. Haddow and S. Weinhouse. New York (Academic Press Inc.).
COX, D.—(1966) *Cancer, N.Y.*, **19**, 1217.
CURCIO, S.—(1966) *Archo Ostet. Ginec.*, Suppl., **71**, 139.
CURCIO, S. AND SARTORI, R.—(1966) *Archo Ostet. Ginec.*, **71**, 423.
ENTERLINE, H. T. AND ARVAN, D. A.—(1967) *Cancer, N.Y.*, **20**, 1746.
FISCHER, P., GOLOB, E. AND HOLZNER, J. H.—(1966) *Z. Krebsforsch.*, **68**, 200.
FORD, C. E. AND CLARKE, C. M.—(1963) *Canadian Cancer Conference*, **5**, 129.
GOH, K., SWISHER, S. N. AND TROUP, S. B.—(1963) *Archs intern. Med.*, **114**, 439.
DE GROUCHY, J., DE NAVA, C. AND BILSKI-PASQUIER, G.—(1965) *Nouv. Revue fr. Hémat.*, **5**, 69, 565.
DE GROUCHY, J., DE NAVA, C., BILSKI-PASQUIER, G., ZITTOUN, R. AND BERNADOU, A.—(1967) *Annls. Génét.*, **10**, 43.
HUNGERFORD, D. A. AND NOWELL, P. C.—(1962) *J. natn. Cancer Inst.*, **29**, 545.
KATAYAMA, K. P. AND JONES, H. W.—(1967) *Am. J. Obstet. Gynec.*, **97**, 978.
KOSS, L. G. AND DURFEE, G. R.—(1961) 'Proliferative disorders and tumors of the endometrium. *In* 'Diagnostic Cytology and its Histopathological Basis'. Philadelphia (J. B. Lippincott Company), Chapter 9, Part 1.
LAMB, D.—(1967) *Br. med. J.*, i, 273.
LEJEUNE, J., BERGER, R., HAINES, M., LAFOURCADE, J., VIALATTE, J., SATGE, P. AND TURPIN, R.—(1963) *C. r. hebd. Séanc. Acad. Sci., Paris*, **256**, 1195.
LEJEUNE, J., BERGER, R. AND RÉTHORÉ, M. O.—(1966) *C. r. hebd. Séanc. Acad. Sci., Paris*, **263**, 1880.
LUBS, H. A. AND KOTLER, S.—(1967) *Ann. intern. Med.*, **67**, 328.
MILES, C. P.—(1967*a*) *Cancer, N.Y.*, **20**, 1253.—(1967b) *Cancer N.Y.*, **20**, 1274.
PAULETE-VANRELL, J. AND COMACHO DE OSORIO, O.—(1966) *Actas Ginecotocologicas*, **20**, 298.
SANDBERG, A. A., ISHIHARA, T. AND CROSSWHITE, L. H.—(1964) *Blood*, 24, 716.
SANDBERG, A. A., ISHIHARA, T., KIKUCHI, Y. AND CROSSWHITE, L. H.—(1964) *Ann. N.Y. Acad. Sci.*, **113**, 663.
SPEED, D. E. AND LAWLER, S. D.—(1964) *Lancet*, i, 403.
WAGNER, D., RICHART, R. M. AND TERNER, J. Y.—(1967) *Cancer, N.Y.*, **20**, 2067.
WAKONIG-VAARTAJA, R.—(1962) *Br. J. Cancer*, **16**, 616.—(1963) *Aust. N.Z. J. Obstet. Gynaec.*, **3**, 170.
WAKONIG-VAARTAJA, R. AND KIRKLAND, J. A.—(1965) *Cancer, N.Y.* **18**, 1101.
WEINSTEIN, A. W. AND WEINSTEIN, E. D.—(1963) *New Engl. J. Med.*, **268**, 253.
WINKLESTEIN, A., SPARKES, R. S. AND CRADDOCK, C. G.—(1966) *Blood*, **27**, 722.